# Digital Acceptance and Commitment Therapy for Lifestyle Change in Overweight Pregnant Women: A Feasibility Pilot Study

**DOI:** 10.3390/bs16040585

**Published:** 2026-04-14

**Authors:** Anna Elena Nicoletti, Michele Tonelli, Barbara Purin, Silvia Rizzi

**Affiliations:** Digital Health Research, Centre for Digital Health & Wellbeing, Fondazione Bruno Kessler, Via Sommarive 18, 38123 Trento, Italysrizzi@fbk.eu (S.R.)

**Keywords:** ACT, well-being, pregnancy, overweight, eHealth, mHealth, development, usability, user-centred design

## Abstract

Overweight and obesity during pregnancy are associated with increased maternal and neonatal risks, yet scalable interventions addressing the psychological processes underlying health behaviours remain limited. This study describes the development and formative evaluation of DEMETRA, a chatbot delivering an Acceptance and Commitment Therapy (ACT)-informed intervention to promote healthier lifestyles in pregnant women. In line with Phase 1 of the Obesity-Related Behavioral Intervention Trials framework, a multidisciplinary team developed a six-session digital program delivered via a rule-based virtual assistant. A mixed-methods design was employed to assess acceptability, usability, and perceived relevance among a heterogeneous stakeholder sample. Sixteen stakeholders (psychologists, communication experts, nutritionists, clinicians, and non-overweight, expectant women or those who had recently delivered) participated in iterative testing; 15 completed quantitative measures (Semantic Differential scales, uMARS, BUS-11) and 16 completed semi-structured interviews. Non-parametric analyses indicated significantly positive evaluations across most communication and content domains, particularly clarity and language appropriateness, whereas session duration and several engagement-related dimensions did not significantly differ from neutrality. Qualitative findings confirmed strengths in clarity, non-stigmatising tone, and multimedia support, while identifying limited personalisation and message pacing as key areas for refinement. Overall, findings provide formative evidence that ACT-informed principles can be translated into a chatbot-delivered antenatal program and highlight concrete priorities for optimisation (e.g., personalisation and message pacing). Because end-user testing did not include overweight/obese pregnant women and the sample was small and heterogeneous, conclusions regarding acceptability/feasibility in the intended clinical population remain preliminary; the results primarily support iterative refinement and subsequent proof-of-concept testing in the target group.

## 1. Background

Overweight and obesity during pregnancy represent a major public health concern, as they are associated with increased risks of gestational diabetes, hypertensive disorders, cesarean delivery, and adverse neonatal outcomes (Almutairi et al., 2024; Brunner et al., 2025). Moreover, excess weight in pregnancy often persists postpartum and contributes to long-term maternal obesity and metabolic complications (Catalano & Shankar, 2017). Interventions targeting nutrition and physical activity have shown some effectiveness in mitigating these risks by preventing excessive gestational weight gain (Shieh et al., 2018; Kurnaz & Karaçam, 2025), even when delivered in a mobile health (m-Health) format (Sari et al., 2025).

Yet the implementation of interventions for pregnant women remains challenging due to, among other factors, low adherence, time constraints, and limited access to in-person programs (Bowden et al., 2023; Ernst et al., 2025; Lelorain et al., 2024).

In recent years, digital health technologies, including mobile applications and conversational agents (virtual assistants), have gained increasing attention as scalable tools to deliver psychological interventions (Conrad, 2024; Torous et al., 2025). Virtual assistants can provide real-time guidance, foster engagement, and overcome barriers such as stigma, geographic distance, or lack of specialized providers (Conrad, 2024; Adler & Van Brunt, 2025). Early studies suggest that such technologies may enhance user adherence and satisfaction, particularly in vulnerable populations such as pregnant women.

Acceptance and Commitment Therapy (ACT) has emerged as a transdiagnostic behavioral approach that has proven effective in promoting psychological flexibility and sustainable health behavior change (Dindo et al., 2017; Hayes et al., 2013; Yıldız, 2020). The ACT endeavors to cultivate psychological flexibility, which is defined as the capacity to sustain present-moment awareness while acting in accordance with personally valued objectives, even in the presence of challenging thoughts, emotions, or bodily sensations. The foundational processes of ACT encompass acceptance, cognitive defusion, mindfulness, and values-based action. Collectively, these processes assist individuals in reducing experiential avoidance and engaging in health-promoting behaviors, making this approach particularly relevant during pregnancy, a period often characterized by emotional and physical challenges that may interfere with adherence to lifestyle recommendations.

A growing body of literature indicates that ACT-based interventions are effective in supporting weight management, physical activity, and dietary improvements in non-pregnant populations (Iturbe et al., 2022; Chew et al., 2023; Pitil & Ghazali, 2024; Schumacher et al., 2025). Concurrently, digital and mobile health interventions have demonstrated potential in enhancing accessibility and engagement in behavioral interventions, including in perinatal contexts (Waters et al., 2020; Howard et al., 2023; Ashton et al., 2025). However, despite these advances, there is a paucity of research on the feasibility and acceptability of ACT-based interventions specifically tailored to overweight and obese pregnant women, particularly when delivered through conversational agents such as chatbots. Addressing this gap is imperative to inform the development of scalable, psychologically informed interventions targeting health behaviors during pregnancy.

### 1.1. An Acceptance and Commitment Therapy Intervention to Promote Healthier Behaviors and Lifestyles in Overweight Pregnant Women

Interest in the development of ACT-based, digital interventions is growing. ACT-based digital interventions have already been developed for patients suffering from chronic health conditions (Gentili et al., 2021; Levin et al., 2025; Herbert et al., 2022), depression (Lappalainen et al., 2015; Apolinário-Hagen et al., 2024), anxiety (Tobias et al., 2022; Kelson et al., 2019), distressed university students (Levin et al., 2020), and for stress management in the general population (Fietta et al., 2024; Klimczak et al., 2023). ACT interventions aimed at either pregnant women (Howard et al., 2023) or overweight and obese populations have also been developed (Schumacher et al., 2025), even in a digitalized format, with promising results (Ashton et al., 2025; Levin et al., 2021). Yet, no attempt has been made regarding the development of such an intervention for overweight or obese pregnant women, even though they are a growing and at-risk population (Kent et al., 2024).

Different guidelines for the care of pregnant, overweight or obese women have been developed, and while some issues remain debated (e.g., optimal Gestational Weight Gain, National Institute for Health and Care Excellence (NICE), (NICE, 2025)), there is an agreement regarding the importance of physical activity and healthy eating for managing weight related risks for both the mother and the child (Ahmad et al., 2025; Jangra et al., 2025). Therefore, we sought to develop a psychoeducational and motivational intervention rooted in ACT to support pregnant women in their struggle to adopt a healthier lifestyle (Dencker et al., 2016; Jelsma et al., 2016), as well as to provide healthcare workers with a low-cost, scalable intervention to support their efforts.

The content architecture of the digital chatbot intervention was informed by two primary resources: The diet trap (Lillis et al., 2014) and Healthy habits sucks (Lee-Baggley, 2019). The former has been successfully employed in interventions for overweight and obese adults, demonstrating benefits in reducing weight-related self-stigma and promoting healthy behaviors (Potts et al., 2022). While we are not aware of empirical evaluations of interventions directly based on Lee-Baggley (2019), the inclusion of content from both sources was guided by the characteristics and needs of our target population. In particular, since intentional weight loss is not recommended during pregnancy (NICE, 2025), the broader behavioral focus of Lee-Baggley (2019) was deemed especially suitable to avoid inadvertently over-emphasizing weight reduction. Lastly, in light of previous results highlighting the potential of using the ACT matrix (Polk & Prevedini, 2017) for the development of digitalized ACT interventions (Levin et al., 2017; Krafft et al., 2019), we also employed it. To guide the inclusion of this tool in the intervention, we relied primarily on Polk and Prevedini (2017).

The material obtained through the extraction and reformulation of the content from the aforementioned sources into concise, conversational modules suitable for chatbot delivery covers core ACT concepts—namely acceptance, cognitive defusion, present-moment awareness, values clarification, committed action and more—with a focus on the domains of nutrition and physical activity during pregnancy. The said content was developed not as a substitute for information on the importance of physical activity and healthy nutrition during pregnancy, but as motivational and psychological support for women seeking to adopt healthier habits.

### 1.2. Present Research

The intervention’s objective is to enhance psychological flexibility in women through ACT-based exercises to promote healthy lifestyles.

The content is delivered through a meticulously designed step-by-step process, in order to promote self-awareness, acceptance, and normalisation of internal states, stress management, and possibly promote overall improvement in psychological well-being. Once perfected based on the results of the current study, this ACT intervention will be fully available to users through digital tools. In particular, it will be delivered by a mobile application and guided by a virtual assistant, DEMETRA. The present research aims to assess the prototype of the DEMETRA chatbot, gathering feedback and needs from key stakeholders to further refine the dialogues from the perspectives of usability, accessibility, and acceptability of the chatbot-delivered intervention.

## 2. Materials and Methods

The design and development of the chatbot DEMETRA followed the recommendations of the Centre for eHealth Research and Disease Management, which suggests a multidisciplinary approach coupled with continuous and systematic evaluation throughout to improve uptake and impact of eHealth technologies (van Gemert-Pijnen et al., 2011).

The ACT intervention program is developed iteratively, following the Obesity-Related Behavioral Intervention Trials (ORBIT) model (Czajkowski et al., 2015), which depicts the pathway to translate behavioral science discoveries into behavioral treatments ready for efficacy testing.

**Figure 1:**
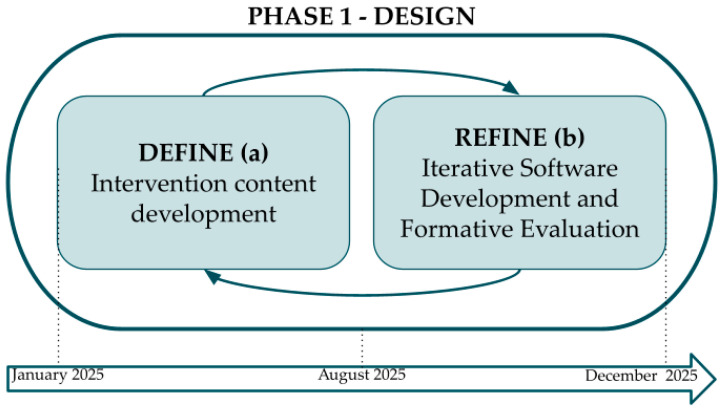
Development and iterative processes of the ACT-based intervention (Phase 1 of the ORBIT model—Design).

The multidisciplinary project team, comprising experts in psychology, eHealth research, and communication, held biweekly meetings during the design and development phase. A user-centred design approach ensured continuous involvement of patients, healthcare providers, and security specialists. Development followed an iterative process through (a) intervention content development (identified and adjusted from the reference material) and (b) iterative software development of a prototype and formative evaluation. The development and iterative processes of the ACT-based intervention are shown in Figure 1.

The development of the intervention’s materials was led by one of the authors (AEN), a researcher and psychologist, in collaboration with a multidisciplinary team of psychologists, ACT experts, nutritionists, communication experts, and information technology experts. The team held recurrent meetings to revise the materials as they were developed.

To identify key themes and issues to address in the intervention, both psychologists and non-psychologists team members were required to read Lillis et al. (2014), Lee-Baggley (2019) and Schoendorff et al. (2014) to familiarize themselves with ACT’s approach to health and dietary behaviors. Group discussions coordinated by one of the ACT experts were held to make sure that there was a shared understanding of the chosen approach. Lillis et al. (2014) and Lee-Baggley (2019) were chosen for their relevance and being targeted to the general population, so as to inform the team’s communication style choices, as well as helping in identifying key themes within the health behaviors domain.

### 2.1. Intervention Content Development

Adapting the reference material into a digital format began with a comprehensive review of both the literature and the original manuals. Once the week-by-week thematic structure was defined, a team of psychologists with expertise in communication adapted the material into a chatbot-based, individualised digital format. The process began with the development of the chatbot’s dialogue scripts, followed by the creation of multimedia resources (videos, audio, and images) to convey key information in a more accessible, engaging format for women than text alone.

The initial intervention content was drafted and subsequently refined through iterative cycles, resulting in a six-session program delivered electronically via text, video, and images. Table 1 outlines the six sessions and their respective topics. The intervention is reported in accordance with the Template for Intervention Description and Replication (TIDieR) checklist to enhance replicability (Hoffmann et al., 2014); a detailed TIDieR table covering both the intended six-week intervention and the accelerated prototype testing procedure is provided in Supplementary Materials (Table S1).

The intervention was designed so that DEMETRA—the chatbot—always initiates the interaction. At this stage, users cannot submit specific queries; instead, the chatbot delivers information and prompts self-reflection through targeted questions. User responses are not analysed in the current phase, as this lies outside the scope of the study.

The intervention is scheduled to span six weeks. Each session lasts approximately 15 min. On the designated day, DEMETRA provides the participant with psychoeducational material; at the end of each session, participants are given instructions for an exercise to be completed during the subsequent week. For the formative prototype evaluation, content was delivered using an accelerated schedule (one session every 48 h over two weeks; average session duration ~15 min) to allow timely feedback across modules.

### 2.2. Iterative Software Development and Formative Evaluation

Adapting ACT interventions through the implementation of the DEMETRA chatbot offers a novel approach to delivering psychological support. Users can participate in comprehensive and effective interventions through personalised sessions. In the initial session, users are prompted to choose one day of the week on which they wish to be contacted by DEMETRA and their preferred time slot (morning, afternoon, or evening). Based on user responses, DEMETRA provides tailored content—for example, DEMETRA asks whether the user would like to review the previous session, whether they prefer to complete a specific exercise immediately or later, and so on, with the dialogue branching accordingly.

At this early stage of development, we decided to adopt the platform Landbot.io for intervention delivery. Landbot.io is a platform that allows for the deployment of rule-based chatbots via WhatsApp, removing the need for the development of proprietary software to do so, greatly reducing barriers to and costs of testing.

In the first iteration, conducted in August 2025, two psychologists and two communication experts tested a preliminary prototype and provided feedback to ensure the intervention program was logically structured. At this co-design stage, the involvement of experienced professionals, alongside end users, was considered crucial. For this reason, in a subsequent study (Test 1), two additional psychologists, three communication experts and three nutritionists were involved to test the low-fidelity prototype. Later, end users were also engaged in the process (Test 2), including four women and four clinicians. This study was conceived as an early-stage formative evaluation (ORBIT Phase 1—Design) of an ACT-informed chatbot prototype. Accordingly, we prioritised feedback on comprehensibility, interaction flow, tone, and perceived appropriateness of the content while minimising potential burden and risk in initial testing. For this reason, we recruited a convenience sample of women who were pregnant or within 12 months postpartum, without medical or psychological complications and with pre-pregnancy BMI < 25, alongside clinicians and other professionals. We acknowledge that this sampling strategy limits conclusions regarding acceptability and feasibility in the intended end-user group (overweight/obese pregnant women) and therefore the findings are interpreted primarily as guidance for iterative refinement prior to target-group evaluation. Women within 12 months postpartum were included to obtain feedback from individuals with recent lived experience of pregnancy-related lifestyle challenges.

Overall, a multistage evaluation of the DEMETRA prototype was conducted between October and December 2025, involving 16 participants.

#### 2.2.1. Study Aims: Quantitative and Qualitative Components

In accordance with ORBIT Phase 1 (Design), the primary objective of this formative mixed-methods study was to assess usability, acceptability, and perceived relevance of the ACT-based intervention delivered via the DEMETRA chatbot within a heterogeneous stakeholder sample, in order to inform refinement prior to testing in the intended clinical population. In line with ORBIT’s emphasis on iterative optimization of intervention components, the quantitative component concentrated on early indicators of user experience and perceived value. User evaluations were collected using standardised 5-point Likert-scale instruments, and median scores were compared against a predefined benchmark corresponding to the scale midpoint (3/5). This was operationalised as the minimum threshold of acceptability. This approach enabled the research team to ascertain whether the prototype attained a satisfactory level of usability and perceived quality, sufficient to inform subsequent refinement and future testing phases.

In line with the principles of implementation research, the concepts of feasibility and acceptability were conceptualised following Proctor et al. (2011). The term ‘feasibility’ refers to the extent to which an intervention can be successfully used or carried out within a given setting. In contrast, ‘acceptability’ reflects the perception among stakeholders that an intervention is agreeable, satisfactory, or appropriate. In the present study, these constructs were operationalised through quantitative and qualitative indicators of usability, user experience, and perceived relevance.

The methodological approach adopted in the present study is consistent with prior feasibility and acceptability studies of digital psychological interventions. In particular, previous research on chatbot-delivered and digital self-help interventions has emphasised the significance of integrating quantitative and qualitative data to facilitate the development and refinement of early-stage interventions. This encompasses recent feasibility protocols and formative evaluations of digital ACT-based and mindfulness-based interventions, along with associated studies conducted within the context of our research. These studies similarly underscore the significance of triangulating user experience measures with in-depth qualitative feedback prior to efficacy testing (Fietta et al., 2024; Nicoletti et al., 2025).

The qualitative component provided a deeper, contextualised understanding of how users experienced the intervention and interacted with the chatbot, thereby complementing the quantitative findings. In particular, qualitative data were utilised to investigate the perceived clarity, tone, and appropriateness of the chatbot’s communication style; the structure, pacing, and burden of the sessions; the perceived relevance of the content to the perinatal period; and the extent to which the intervention was experienced as personalised. In accordance with the formative aims of ORBIT Phase 1, qualitative feedback was also used to identify implementation barriers, unmet user needs, and actionable suggestions for improvement. The integration of quantitative and qualitative data was intended to inform iterative refinement of both intervention content and delivery mechanisms, with the objective of optimising the DEMETRA chatbot before advancing to later phases of intervention testing.

#### 2.2.2. Variables Identification

Although acceptability and implementation frameworks can guide measurement selection, this formative ORBIT Phase 1 evaluation was not prospectively designed around the Theoretical Framework of Acceptability or RE-AIM; for transparency, we provide a post hoc mapping of collected indicators to these frameworks in Supplementary Table S2 (Sekhon et al., 2017; Kessler et al., 2013).

The quantitative evaluation focused on key domains relevant to chatbot-based digital interventions, including communication, session structure, materials, content, engagement, functionality, aesthetics, information quality, subjective quality, and perceived impact. The communication session structure, materials, and content were assessed using an ad hoc Semantic Differential scale (Osgood et al., 1957), developed to capture participants’ perceptions of tone, clarity, and overall communicative effectiveness. The Semantic Differential questionnaire was developed for this formative evaluation to capture early-stage perceptions of (i) communication style and tone, (ii) module structure (including perceived session duration/burden), (iii) materials (e.g., videos and infographics), and (iv) content quality and clarity—domains that were central to the study aims and not fully covered by generic app- or chatbot-usability instruments. Following the semantic differential approach (Osgood et al., 1957), each item consisted of a bipolar adjective pair rated on a 5-point scale (1–5), with the theoretical midpoint (3) representing a neutral evaluation. Item wording and domain coverage were defined by the multidisciplinary team (psychology and communication experts in particular) and refined through iterative prototype testing to ensure clarity, appropriateness, and sensitivity to weight-related themes in pregnancy. Composite dimension scores were computed by averaging item ratings within each sub-domain (see Appendix A for item-level content). Higher scores indicate a more favourable evaluation. Content validity was supported through iterative multidisciplinary expert review (psychology, ACT, communication, and eHealth), with revisions applied until consensus on clarity and domain coverage was reached.

The remaining domains were evaluated using validated instruments, namely the User Version of the Mobile Application Rating Scale (uMARS; Stoyanov et al., 2016) and the Italian version of the Chatbot Usability Scale (BUS-11; Borsci et al., 2023).

The uMARS is a tool specifically designed for the evaluation of mobile health applications (mHealth), with an Italian version demonstrating construct validity and a stable multidimensional structure (Morselli et al., 2021). The instrument is composed of 20 items that evaluate engagement, functionality, aesthetics, information quality, subjective quality, and perceived impact. In line with the objectives of the present study and with a view to minimising overlap with chatbot-specific usability measures, the functionality and aesthetics subscales were excluded. Given the multidimensional structure of the uMARS, internal consistency was assessed separately for each retained subscale. In the present sample, Cronbach’s alpha coefficients indicated good internal consistency across subscales (engagement α = 0.88; information α = 0.74; subjective quality α = 0.83; perceived impact α = 0.92), in line with previous validation studies reporting high reliability (e.g., Morselli et al., 2021).

While the uMARS provides a broad assessment of the quality of a mHealth intervention, the BUS-11 complements this evaluation by focusing specifically on conversational aspects of the interaction. The BUS-11 is an 11-item scale designed to assess user satisfaction with chatbot interactions. It encompasses accessibility and the quality of functions, the quality of conversation and information, perceived privacy and security, and response time. The scale has been validated in multiple languages, including Italian, with evidence of construct validity, convergent validity with other usability measures and high internal consistency in previous validation studies (Borsci et al., 2023). In the present sample, the BUS-11 showed acceptable internal consistency (Cronbach’s α = 0.78). Its main advantage lies in its specificity for conversational agents, allowing the assessment of interactional qualities that are not captured by app-quality instruments such as the uMARS, while maintaining a low respondent burden suitable for early-stage evaluations. Given the small sample size (*n* = 15), internal consistency estimates should be interpreted with caution.

Subsequent to the completion of the quantitative measures, a semi-structured interview was conducted to explore participants’ experiences in greater depth, with particular attention to perceived strengths, limitations, and suggestions for improvement (see Appendix B for details). The framework method (Gale et al., 2013) was employed to analyse the qualitative data, with a view to identifying recurrent patterns and themes that complemented and contextualised the quantitative findings. The employment of a mixed-methods approach resulted in a comprehensive formative evaluation of the DEMETRA prototype, thereby providing direction for subsequent iterations.

#### 2.2.3. Procedure

Convenience sampling was deemed appropriate for the research purpose; therefore, participants were recruited among direct acquaintances via word of mouth, and the aim of the study was clearly stated. Participants were then contacted via email to receive the informed consent form, which included all relevant information. Only once the researchers received the signed consent form were people considered enrolled in the study.

After the enrollment, participants received detailed instructions via email on how to interact with the DEMETRA chatbot, as well as additional information regarding the testing procedure. At the same time, they received the opening session of the intervention on WhatsApp.

For the next two weeks, participants received the intervention every 48 h, allowing them a reasonable interval to interact with the chatbot at their own pace and on their own schedules. The sessions lasted an average of 15 min and provided the user with readable and watchable content. They were required to interact by offering close- and open-ended questions to proceed with the conversation and foster both engagement and self-reflection. The answers provided by participants were neither collected nor analyzed in this prototyping phase, as it was not required by the aim of the study.

Once the testing period ended, participants received the questionnaire via email and were invited to schedule an appointment for the final interview to further report on their expectations, preferences, and concerns regarding the solution tested. The interview questions were designed ad hoc for our study, focusing on topics relevant to the evaluation of the chatbot. The inclusion criteria for participating in the interview were: having completed the prototype test and providing consent to participate in this additional phase. The researcher conducted 16 interviews, which were then analysed and processed using qualitative tools. The conductor initially provided a brief introduction to the interview objectives. Then, participants were asked to answer a series of semi-structured questions regarding their expectations and preferences for using the chatbot. Interviews lasted approximately 15 min and were audio-recorded to facilitate later analysis. All data were collected in Italian and pseudonymized, with participants’ informed consent. Confidential audio recordings of semi-structured interviews were used for data analysis, and subjects were identified only by numeric codes. At the study’s conclusion, participants can request the research outcomes from the research manager.

According to Italian Law No. 3/2018, formal ethical approval was not required for this study, as it focused on the usability evaluation of a prototype digital intervention and did not involve clinical treatment or the collection of sensitive health data. 

Despite the inclusion of pregnant and postpartum women in the study, participants were not recruited as patients and were required to meet low-risk criteria. These criteria included the absence of medical or psychological complications and a pre-pregnancy BMI of less than 25. The study’s scope was confined to the evaluation of a non-clinical prototype, precluding the assessment of health-related or behavioral outcomes. 

In light of these characteristics, the study was considered low-risk. Notwithstanding, all procedures were conducted in accordance with ethical principles, including voluntary participation, informed consent, and data protection.

Participants were not rewarded for their participation.

#### 2.2.4. Data Analysis

The open statistical software Jamovi version 2.3.28.0 (jamovi—open statistical software for the desktop and cloud, s.d.) was used to analyze the quantitative data from the Semantic Differential, the BUS-11 and the uMARS. Considering the numerosity of the sample, we used non-parametric tests: the Wilcoxon signed-rank test (W) was used instead of the one-sample *t*-test (King & Eckersley, 2019; Tomczak & Tomczak-Łukaszewska, 2014). Rank-biserial correlation (r) was reported to indicate effect size (Tomczak & Tomczak-Łukaszewska, 2014). A nominal alpha level of 0.05 was used, but results are interpreted cautiously given the exploratory nature and multiplicity of tests.

Given the number of one-sample tests across multiple domains, analyses were treated as exploratory and interpreted with emphasis on effect sizes and convergence with qualitative findings. No formal multiplicity correction was applied in this formative phase.

Qualitative data from the interviews were analyzed using the framework method (Gale et al., 2013), adopting a mainly deductive approach. Prior to analysis, an analytical framework was defined by the research group, identifying thematic categories and codes based on the key variables of the study and the topics guiding the interview questions.

The analysis of each interview began with a familiarization phase, during which audio recordings and transcripts were reviewed to ensure an overall understanding of the content. This was followed by an indexing phase, in which the researcher labeled relevant passages according to the predefined codes. Although a formal open coding phase was not conducted, instances in which the existing coding framework appeared insufficient were noted during analysis.

Following indexing, data were charted into the framework matrix, distinguished according to participant type for interpretation.

This study is a formative mixed-methods prototype evaluation (ORBIT Phase 1—Design) and is not a randomised pilot trial. Accordingly, SPIRIT and the CONSORT extension for randomised pilot and feasibility trials are not fully applicable to the present manuscript; these guidelines will be adopted for the protocol and reporting of subsequent randomised pilot and effectiveness studies.

## 3. Results

The pilot evaluation was conducted with 16 participants, including two psychologists, three communication experts, three nutritionists, four mothers, and four clinicians. Of these, 15 responded to the questionnaire (only one mother did not respond), and 16 participated in the semi-structured interview.

The mean age of the subjects who completed the questionnaire was 35.3 years (SD = 7.92, ranging from a minimum of 26 to a maximum of 50 years).

Given the very small and heterogeneous sample, quantitative findings are presented primarily to support formative and descriptive interpretation. To enhance interpretability, we additionally report descriptive statistics stratified by stakeholder type, distinguishing end users (women; *n* = 3) from professionals/clinicians (psychologists, clinicians, nutritionists, and usability experts; *n* = 12). Because subgroup sizes are small, these stratified results are intended to provide context rather than to support between-group inferential comparisons.

### 3.1. Quantitative Results

The use of a semantic differential–based questionnaire makes it possible to examine respondents’ average positioning in relation to the four macro-variables under investigation. More specifically, regarding the sub-variables, the Wilcoxon analysis revealed several statistically significant results, as reported in Table 2. Given the small sample size, *p*-values are interpreted cautiously; emphasis is placed on descriptive patterns and effect sizes, complemented by qualitative triangulation.

Using a 1–5 scale with a theoretical median of 3 (neutral), participants’ ratings were overall skewed toward the positive pole. Wilcoxon one-sample tests showed statistically significant deviations above the neutral value for 8 out of 9 dimensions (*p* < 0.05) supporting a systematic shift toward favorable evaluations: empathy and listening (W = 103.5, *p* = 0.001, r = 0.971), smoothness and fluidity (W = 79.5, *p* = 0.015, r = 0.747), chatbot interaction (W = 93.5, *p* = 0.011, r = 0.781), and lexicon (W = 105.0, *p* < 0.001, r = 1.000). Similarly, content-related and multimedia dimensions were significantly above neutrality, including videos (W = 105.0, *p* = 0.001, r = 1.000), infographics (W = 89.0, *p* = 0.003, r = 0.956), overall content evaluation (W = 115.5, *p* = 0.002, r = 0.925), and content clarity (W = 91.0, *p* = 0.001, r = 1.000). The strongest descriptive and inferential signals were observed for content clarity and lexicon (highest means, medians at 4.0, and very large effect sizes), suggesting particularly favourable perceptions of comprehensibility and language appropriateness; however, the concentration of medians at 4.0 and several very large r values (≈1.0) may also be consistent with a degree of ceiling tendency in responses. Variability patterns further nuanced these findings: while most items showed moderate dispersion, smoothness and fluidity displayed the highest variability (SD = 0.99), indicating more heterogeneous experiences despite a positive average. In contrast to the generally positive pattern, session duration was the only dimension that did not significantly differ from neutrality (M = 3.23, SD = 0.75; Md = 3.00; W = 31.0, *p* = 0.328, r = 0.378), aligning with its lower central tendency values and suggesting that perceptions of time allocation remained closer to neutral. Finally, given that multiple one-sample tests were conducted (9 outcomes), the overall pattern remains strongly positive, but the small sample size and the number of comparisons warrant cautious interpretation of the comparatively higher *p*-values (e.g., for smoothness and chatbot interaction), whereas the lowest *p*-values (e.g., lexicon, clarity, videos, infographics, content evaluation, empathy) indicate particularly robust departures from neutrality.

Descriptively, women tended to rate smoothness and fluidity, lexicon, fluidity, and session duration slightly higher than professionals/clinicians, whereas professionals/clinicians provided higher ratings for videos, infographics, and content evaluation and content clarity; both groups showed similarly high ratings on chatbot interaction and empathy.

BUS-11 results (*n* = 15) also supported a positive evaluation relative to the neutral benchmark (3) as shown in Table 3.

Wilcoxon signed-rank tests showed significant deviations above neutrality for Accessibility (W = 115.0, *p* = 0.002, r = 0.92), Quality of features (W = 116.5, *p* = 0.001, r = 0.94), Privacy and security (W = 61.5, *p* = 0.009, r = 0.86), and Response time (W = 105.0, *p* < 0.001, r = 1.00). Conversation quality was also significantly above neutrality (W = 97.0, *p* = 0.037, r = 0.62), but it showed the lowest central tendency and the smallest effect size among the dimensions, suggesting comparatively greater room for improvement. Across dimensions, effect sizes were predominantly large, indicating that observed differences from the neutral point were not only statistically detectable but also substantively meaningful in this sample.

Descriptively, women tended to rate accessibility and response time slightly higher than professionals/clinicians, whereas professionals/clinicians tended to provide higher ratings for privacy and security and, to a lesser extent, conversation and information quality; both groups showed similarly high ratings for functionality quality.

The uMARS evaluated the respondents’ average positioning in four key dimensions. Detailed results from the Wilcoxon tests are summarised in Table 4.

Information (*n* = 13; 86.67%) was rated highly and showed a significant positive deviation from neutrality (W = 91.0, *p* = 0.002, r = 1.00), suggesting that participants perceived the informational content as particularly strong. By contrast, Engagement (*n* = 15) did not significantly differ from the neutral benchmark (W = 66.0, *p* = 0.160, r = 0.45), nor did Subjective quality (W = 52.5, *p* = 0.089, r = 0.59) or Perceived impact (W = 90.0, *p* = 0.093, r = 0.50). Although these latter domains showed descriptively above-neutral central tendencies (means and medians > 3), the inferential tests did not provide sufficient evidence to conclude that they differed reliably from neutrality in this sample. Notably, the reduced sample size for the Information domain (*n* = 13) should be considered when comparing domains, and the relatively larger SDs for Engagement and Perceived impact (≈0.8) suggest greater heterogeneity in participants’ experiences.

Descriptively, professionals/clinicians tended to rate engagement, information quality, subjective quality, and perceived impact higher than women, with the largest differences observed for engagement and subjective quality; both groups nevertheless showed mid-to-high evaluations overall.

### 3.2. Qualitative Results

A psychologist conducted the qualitative interviews to collect additional information. The interviews were carried out and analysed in line with the predefined thematic variables.

Qualitative themes were broadly consistent across stakeholder groups; however, differences emerged in the emphasis placed on specific aspects of the intervention, reflecting participants’ professional backgrounds. Women more often emphasised experiential aspects of use (e.g., tone, pacing, and perceived personalisation), whereas professionals’ perspectives more often reflected domain-specific priorities (e.g., clinical appropriateness, implementation within care pathways, or user-experience design). Accordingly, women tended to focus on the emotional and interactional qualities of the chatbot, while midwives, nutritionists, and psychologists emphasized the appropriateness and need for psychologically informed interventions to support healthier behaviors. Usability experts, in contrast, primarily focused on user experience.

The insights generated by the qualitative analysis are presented in further detail below.

#### 3.2.1. Set 1: Communication

Overall, participants appreciated the communication style of DEMETRA, albeit some critiques emerged.

While the majority of participants found the communication style of the chatbot to be fit for the themes and the target audience, some found that the emotional qualities of the communication could be ameliorated. In particular, a couple of participants (1 psychologist and 1 expecting mother) pointed out that the responses the chatbot offers after asking the user about their current well-being seem formulaic and impersonal, albeit correct in content. On a more general level, some testers (among which two usability experts) pointed out that it was evident they were talking with a chatbot due to the cadence with which they were requested to provide feedback in order to proceed in the interactions.

Nonetheless, participants found the chatbot to be supportive and motivating, with psychologists, nutritionists and midwives stating that the chatbot touches on many issues that overweight pregnant women face in their efforts to adopt healthier lifestyles and during gestation.

Interacting with DEMETRA was judged to be both easy and seamless, with many appreciating the close-answer format adopted for the majority of the interactions; many also reported cherishing the possibility of asking the chatbot to repeat or further explain concepts within and between sessions, helping the user follow the dialogue with ease.

An issue that arose repeatedly, though, was the speed at which the chatbot sent the messages, often deemed too fast. While this is something that we could not control on Landbot.io, we will address the problem moving forward with the development of DEMETRA.

With regard to the adopted language (and as will be further elaborated in the Section 3.2.4) the overwhelming majority of participants found the communication to be easily understandable and accessible.

#### 3.2.2. Set 2: Module Structure

Consistent with the results from the semantic differentials, participants reported conflicting views on session duration, with some participants suggesting lengthening the sessions, some finding them adequate, and others advocating shortening them. Notably, though, expecting mothers expressed favorable evaluations regarding session durations. Interestingly, one expecting mother suggested that the modalities of content delivery and session duration could be more fit for certain users than for others, pointing in particular to the amount of written information provided (versus audio-visual) as a possible risk for younger mothers. Consistent with this comment, many among those who were critical of module structure pointed out that some messages were too verbose, negatively affecting the user experience and possibly leading users to skim through the contents; suggested solutions to target these issues were either splitting said messages into shorter chunks or substituting them with audio-visual content. To quote a nutritionist on the matter: “thinking about my own experience with patients, I noticed that whenever you give lots of written informational material to them, they tend to skip the reading… I’m afraid that once the interaction slows down and the user has to stop and read longer passages, you might lose their attention. I don’t know if it is feasible, but I would consider adopting podcast-like audio messages instead of written texts in those passages”.

Instead, the inclusion of exercises was unanimously deemed a positive aspect of the modules, with two usability experts suggesting to further develop this aspect to ensure higher engagement.

Personalization emerged as a lacking aspect of the intervention (particularly for usability experts): most participants found the pre-structured nature of the dialogue to be evident, with few aspects of the interaction perceived as truly tailored to them. Participants nevertheless reported feeling able to influence the interaction, primarily by determining which conversational branch the chatbot followed.

Lastly, the usage of images and videos was highly appreciated (further developed in the Section 3.2.3 below), with one expecting mother suggesting providing ever more visual content throughout the intervention.

#### 3.2.3. Set 3: Materials

As anticipated in the previous section, the images and videos provided by the chatbot were unanimously appreciated, with many users identifying them as their favorite aspect of the intervention. According to the testers, the visual materials were especially useful in providing an immediate rehearsal or summary of the content, thereby increasing its accessibility and memorability. Notably, psychologists, nutritionists, and midwives alike reported finding the materials highly communicative and clarifying with regard to the concepts being presented, and thus considered them a valuable support for end users.

Nevertheless, two testers reported that the videos’ voice-overs sounded too artificial and robot-like, while a few others provided suggestions for improving specific pieces of content (e.g., particular videos or images).

#### 3.2.4. Set 4: Content

Feedback on the contents of the intervention was unanimously positive, with testers across all categories expressing their appreciation for how the promotion of healthier behaviors was approached. As previously mentioned, participants found the dialogues and sessions to exhaustively cover themes and issues relevant to the needs of the target audience, with midwives, psychologists and nutritionists alike strongly appreciating the lack of emphasis on weight loss or calorie reduction, which would have been potentially detrimental to both the physical and mental health of overweight pregnant women and their unborn children. All the testers found the content to be socially acceptable and sensitive to the target population’s needs.

Content clarity was one of the most appreciated features of the intervention, considered by testers to be resulting from both the adopted language and the ever-present possibility to ask for further clarification. This being said, there were users reporting difficulties with some of the earlier exercises, noting that they struggled to understand whether their instructions and open questions were referring to mental health, physical health or both.

#### 3.2.5. Insights into the Effectiveness of the Intervention

While during the interviews we did not explicitly ask for feedback on the perceived or expected effectiveness of the intervention, participants spontaneously commented on the matter or touched on it when asked about the ideal moment for delivering the intervention.

Generally, testers were optimistic regarding the possible effects of interacting with DEMETRA, highlighting the motivational and supportive features of the chatbot, even though intrinsic motivation was commonly brought up as a perceived requirement for the intervention to be effective. Most interestingly, though, professionals—midwives and nutritionists in particular—expressed highly positive attitudes toward the intervention, welcoming the novel and reportedly needed solution for addressing the psychological and motivational issues users might experience in their effort of adopting healthier lifestyles. Indeed, both categories of professionals reported a general lack of attention to these issues within the healthcare system, despite the increasing relevance and burden associated with weight-related complications during pregnancy. Accordingly, midwives and nutritionists alike considered the intervention potentially effective for users as well as highlighting its value as a supportive tool for their work.

Regarding the optimal timing for delivering the intervention, participants showed a high degree of agreement, generally indicating early pregnancy. Midwives, in particular, consistently identified the beginning of the second trimester (twelfth/thirteenth week) as the most appropriate time. They attributed this to the reduced risk of spontaneous miscarriage, the reduction in first-trimester symptoms, and the perception that patients are more settled and receptive at this point of gestation.

Testers across categories further highlighted the opportunity of capitalizing on the high motivation and interest that expectant mothers can show in early pregnancy.

Lastly, some users suggested continuing the intervention even postpartum.

#### 3.2.6. Overall Assessment

Taken together, the qualitative interviews highlighted a generally positive reception of the intervention across participant categories. Participants consistently appreciated the communication style, clarity of content and use of visual materials, which were widely perceived as supportive, accessible and engaging. Overall, the intervention was judged to be acceptable, understandable and adapt for the target population.

At the same time, though, the analysis revealed several recurring critical points, including limitations in personalization, occasional issues related to message pacing and concerns regarding the length and density of some written messages. While users reported a sense of influence over the interaction, this was primarily associated with navigating predefined conversational paths rather than with content being tailored to individual needs.

Feedback from professionals and potential users alike thus pointed to a combination of strengths and areas for improvement, offering a nuanced picture of the intervention’s current functioning as experienced by different stakeholders.

Table 5 provides quotes from participants reflective of the main positive and negative aspects that emerged from the interviews.

## 4. Discussion

The present formative study examined the acceptability and usability of a chatbot-delivered, ACT-informed intervention designed to address unhealthy eating behaviours and weight-related challenges during pregnancy. The findings indicate that the intervention was perceived as clear, structured, and relevant. Quantitative evaluations of communication and content quality are supported by qualitative feedback, which highlights the comprehensibility and supportive tone of the chatbot. The findings of the study also concur with previous literature in showing the potential of transposing ACT principles in a digital format. While these results provide preliminary insights into the acceptability and usability of the DEMETRA chatbot, it is important to note that they are based on a small, heterogeneous sample and should be interpreted with caution. Concurrently, patterns across engagement-related domains exhibited greater heterogeneity. While the user experience was found to be broadly positive, both quantitative and qualitative data indicated variability in perceived interaction depth, pacing, and personalisation. This convergence of findings is consistent with the objectives of ORBIT Phase 1, in which early-stage evaluations are intended to identify strengths and areas for optimisation rather than to establish effectiveness. The results of the study indicate that the intervention’s conceptual and psychoeducational components are well received, while interactive and adaptive features represent key targets for further refinement.

Feedback from healthcare professionals highlighted a perceived structural gap in routine antenatal care, where guidance on nutrition and physical activity is often provided without systematic attention to the psychological and motivational processes that sustain behavior change. DEMETRA was perceived as addressing this gap by offering structured support focused on acceptance, values, and self-regulation. These components are rarely targeted in standard care pathways.

The findings of this study support the technical and procedural feasibility of chatbot-based delivery of ACT-informed content during pregnancy, and they provide guidance for iterative development that is grounded in empirical evidence. The insights generated through this mixed-methods formative evaluation provide guidance for the purpose of further optimization. Moreover, it is essential to inform decisions regarding progression to subsequent phases of development within the ORBIT framework. However, given the modest sample size and the limited representativeness of the participants, the findings cannot be considered definitive evidence of feasibility or acceptability in the intended clinical population.

### Strengths, Limitations, and Future Directions

The findings of this preliminary formative evaluation suggest that a chatbot-delivered ACT-informed intervention is a promising approach to support weight- and health-related behaviour change during pregnancy. However, results are exploratory and based on a small, heterogeneous, non-target sample; therefore, they should not be generalised to overweight/obese pregnant women. The six-session structure supported fidelity to evidence-based ACT principles while allowing content to be delivered in a concise and accessible format. Digital delivery via chatbot offers clear advantages in terms of scalability, standardisation of content, and potential reach, particularly in contexts where access to specialised psychological or nutritional support may be limited. The intervention was perceived to be clear, well-structured, and relevant to weight-related challenges during pregnancy.

Furthermore, participants indicated that the second trimester of pregnancy was perceived as the most opportune period to engage with the intervention, once both initial, unpleasant symptoms and the risk for spontaneous miscarriage are reduced, and motivation is high. This observation is consistent with prior research emphasising the importance of timing in antenatal behavior change interventions (Waters et al., 2020). In addition, the integration of chatbot-based support with professional guidance, such as nutritional counselling or antenatal care, was proposed by some participants as a particularly effective strategy, as opposed to its provision as a standalone solution.

In accordance with the extant literature on digital health interventions, the present study lends support to the potential of chatbot-delivered programs to promote engagement, provide psychoeducation, and encourage reflection and behaviour change during pregnancy. Participants expressed satisfaction with the systematic and intelligible presentation of content, as well as the intuitive nature of the interaction. Concurrently, the challenges previously identified in the field of chatbot research, including limited flexibility, personalisation, and nuanced responsiveness, were also observed. Reports of repetitive content and constrained interaction pathways reflect ongoing design challenges in balancing automation with individualised support in ACT-based digital interventions.

A significant strength of this study is its participatory and multidisciplinary development process. The involvement of psychologists, ACT experts, nutritionists, communication specialists, and end users throughout the design and formative evaluation phases enhanced the relevance, clarity, and usability of the intervention. Moreover, the integration of quantitative and qualitative methods yielded complementary insights into both measurable aspects of user experience and more nuanced perceptions, consistent with best practices for formative evaluation within the ORBIT framework.

It is imperative to acknowledge the limitations inherent in the interpretation of these findings, which are characteristic of formative evaluations of early-stage digital interventions.

The limited sample size and the high educational level of the participants restrict the generalisability of the results. While the heterogeneity of our sample has allowed us to capitalize on the perspectives brought forth by different stakeholders, an additional and central cause for caution in interpreting our findings is the inclusion criteria adopted for involving new or expectant mothers. Specifically, while having excluded women with BMI > 25 and with no medical or psychological complication lessens ethical concerns regarding participant’ safety, it also limits the scope and the generalizability of our findings to the intended target population. While the generated insights concerning the acceptability and usability of DEMETRA are nonetheless valuable given the different, but converging, perspectives from which they have arisen, we recognize the need for directly including overweight/obese pregnant women moving forward.

In addition, data collection was not prospectively structured around a comprehensive acceptability or implementation framework (e.g., the Theoretical Framework of Acceptability or RE-AIM). While satisfaction and positive appraisal were partially captured through the quantitative usability/app-quality measures and qualitative interviews, attrition and retention across the intended six-week dose could not be meaningfully estimated because the prototype was delivered on an accelerated two-week schedule for formative feedback. Participants identified opportunities for improvement related to personalisation, content variation, and depth of interaction, highlighting the broader challenge of delivering structured, automated interventions while remaining responsive to individual needs and preferences, particularly when addressing sensitive weight-related behaviours during pregnancy. The present study concentrated on the evaluation of usability, acceptability, and perceived impact. Behavioral and clinical outcomes, including alterations in eating behaviors and gestational weight gain, were not the focus of the present study. Finally, the deliverance of the intervention’s prototype in compressed format suggests further caution in interpreting the results, given that, among other things, the accelerated cadence and fixed intervention schedule might have influenced judgements of personalization, as well as possibly generating perceptions of repetitiveness. When considered as a whole, these limitations offer valuable guidance for further refinement and future phases of evaluation.

Future iterations of the DEMETRA intervention should build directly on the formative insights identified in this study. The enhancement of personalisation and flexibility is a key priority, for example through the implementation of more adaptive conversational pathways, increased variability in content delivery, and options for user-driven pacing. Allowing participants to adjust session frequency or revisit selected modules according to their needs may help sustain engagement and reduce perceptions of repetition.

At this developmental stage, DEMETRA relies on structured, rule-based conversational pathways rather than fully adaptive AI-driven dialogue systems. This design choice was intentional and consistent with the formative aims of ORBIT Phase 1, prioritising content fidelity, clarity, and safety over conversational complexity. More adaptive and personalised dialogue features represent a key direction for future iterations.

To guide progression beyond ORBIT Phase 1, we defined a priori criteria for moving to a proof-of-concept evaluation in the intended target group and for subsequent trial phases. Progression will be determined using a Go/Amend/Stop approach incorporating: (i) acceptability/usability indicators (self-report measures and qualitative feedback), (ii) feasibility of study procedures (recruitment, completion of assessments), (iii) objective engagement indicators derived from usage tracking during the six-week delivery, and (iv) safety/technical performance. If these criteria are met, we will proceed to a pilot randomised evaluation to refine procedures and estimate parameters for a subsequent fully powered RCT assessing behavioural and/or clinical outcomes.

Subsequent research should extend the evaluation beyond usability and acceptability to include preliminary behavioural and clinical outcomes, such as changes in eating behaviours, psychological flexibility, or patterns of gestational weight gain, in line with progression to later ORBIT phases focused on refinement and early efficacy testing. Longer-term studies would also allow examination of sustained engagement across different stages of pregnancy. In the planned proof-of-concept study, DEMETRA will be evaluated in the intended target group (overweight/obese pregnant women) using a prospective measurement plan informed by established acceptability and implementation frameworks (e.g., TFA and RE-AIM). Engagement and implementation outcomes will be assessed via objective usage tracking (e.g., module completion, time-on-task, and response/interaction patterns) alongside self-report measures and qualitative interviews. This will enable systematic estimation of reach and retention/attrition across the intended six-week dose, and will better inform progression to later ORBIT phases and subsequent powered trials. Ultimately, it is recommended that subsequent research endeavours encompass larger and more heterogeneous samples, incorporating women from diverse socioeconomic, cultural, and educational backgrounds, in addition to those exhibiting a higher propensity for excessive gestational weight gain. The integration of the chatbot within routine antenatal or nutritional care, whether as a low-intensity standalone intervention or as part of a stepped or blended care model, has the potential to further enhance both impact and trust. Furthermore, it may help to clarify the role of chatbot-based ACT interventions in supporting healthy weight-related behaviours during pregnancy.

## 5. Conclusions

The present study documented the development and formative evaluation of DEMETRA, a chatbot informed by Acceptance and Commitment Therapy (ACT). The purpose of the DEMETRA chatbot is to support overweight and obese women during pregnancy in adopting healthier eating behaviours and lifestyles. In accordance with Phase 1 of the ORBIT framework, the primary objective was not to ascertain efficacy, but rather to evaluate acceptability, usability, perceived relevance, and to identify strengths and areas for refinement prior to subsequent testing.

The findings, when considered as a whole, indicate that the core principles of ACT can be meaningfully and coherently translated into a chatbot-delivered intervention within the antenatal context. DEMETRA was perceived as clear, structured, and appropriate for pregnancy, with particular appreciation for its non-stigmatising, non-weight-loss-centred approach and its focus on values, psychological flexibility, and sustainable health-related behaviours. The utilisation of visual and multimedia materials served to enhance comprehension and accessibility, thereby reinforcing the psychoeducational and motivational objectives of the intervention.

In accordance with the preceding discussion, engagement-related dimensions demonstrated greater variability, and the formative evaluation identified limited personalisation, constrained conversational flexibility, and message pacing as primary targets for optimisation. These findings reflect the known challenges associated with early-stage chatbot-based interventions and underscore the necessity of iterative development to balance standardisation with responsiveness to individual needs, particularly when addressing sensitive weight- and health-related issues during pregnancy.

It is noteworthy that both professionals and potential end users underscored the significance of positioning DEMETRA as a complementary, low-intensity support integrated within routine antenatal or nutritional care, as opposed to a standalone solution. This finding is consistent with the intervention’s designated function as a scalable instrument capable of providing support to healthcare providers while addressing motivational and psychological processes that are frequently inadequately targeted in standard prenatal care.

In conclusion, the present study provides a foundation for future research and development related to the continued advancement of DEMETRA, emphasizing preliminary priorities for optimization. While the study provides insights into usability, acceptability, and perceived relevance, the small, heterogeneous sample limits the extent to which these findings can be generalized. Further testing in the intended target population is required before drawing conclusions about feasibility or effectiveness. While the findings support the promise of translating ACT-informed principles into a chatbot-delivered format and highlight perceived clarity, appropriateness, and non-stigmatising framing, the present evaluation does not establish acceptability or feasibility in the intended clinical population of overweight/obese pregnant women, given the small and heterogeneous sample and the characteristics of the women included. Future research should therefore prioritise proof-of-concept testing in the target group, including objective usage tracking across the intended six-week dose, assessment of preliminary behavioural and clinical outcomes, further enhancement of personalisation and dialogue flexibility, and evaluation of implementation within real-world healthcare settings. Consequently, DEMETRA represents a promising digitally delivered ACT-informed approach to support healthier lifestyles during pregnancy, pending confirmation in the target population.

Overall, the present formative evaluation primarily supports iterative refinement and provides a rationale for subsequent testing in the intended target group, rather than establishing feasibility and acceptability within that clinical population.

## Figures and Tables

**Table 1:** Description of the topics covered week by week.

Week	Topics
Week 0	Brief introduction to DEMETRA and how the chatbot works
Week 1	Biological drivers of unhealthy behaviors; values; factors influencing body weight.
Week 2	The “Fix me-trap” and controlling thoughts and emotions as sources of suffering.
Week 3	Fusion and obedience to thoughts and feelings, cognitive defusion; introduction to the ACT Matrix for value clarification; internal and external obstacles; behavioral avoidance; thoughts and feelings as unavoidable.
Week 4	Recognizing avoidant behaviors and valued actions using the ACT matrix; the myth of the perfect goal-weight.
Week 5	Emotional eating; mindful eating exercise;
Week 6	Using the matrix in daily life; self-compassion vs. self-critique; nine steps to stay on track when pursuing values.

**Table 2:** Results from the Semantic Differential (N = 15).

	Participants, *n* (%)	Values, Mean (SD)	Values, Median (Range)	*W*	*p* Value	r
Empathy and listening	15 (100)	3.68 (0.52)	3.80 (0.14)	103.5	0.001	0.971
Smoothness and fluidity	15 (100)	3.87 (0.99)	4.00 (0.26)	79.5	0.015	0.747
Chatbot interaction	15 (100)	3.72 (0.78)	3.75 (0.20)	93.5	0.011	0.781
Lexicon	15 (100)	4.20 (0.59)	4.00 (0.15)	105.0	<0.001	1.000
Session duration	15 (100)	3.23 (0.75)	3.00 (0.19)	31.0	0.328	0.378
Videos	15 (100)	4.02 (0.59)	4.00 (0.15)	105.0	0.001	1.000
Infographics	15 (100)	3.76 (0.61)	3.67 (0.16)	89.0	0.003	0.956
Content evaluation	15 (100)	3.89 (0.64)	4.00 (0.17)	115.5	0.002	0.925
Content clarity	15 (100)	4.30 (0.70)	4.00 (0.18)	91.0	0.001	1.000

Note. H_a_ μ ≠ 3.

**Table 3:** Results from the BUS-11 (N = 15).

	Participants, *n* (%)	Values, Mean (SD)	Values, Median (Range)	*W*	*p* Value	r
Accessibility	15 (100)	4.20 (0.78)	4.00 (0.20)	115.0	0.002	0.917
Quality of features	15 (100)	4.04 (0.67)	4.00 (0.17)	116.5	0.001	0.942
Conversation quality	15 (100)	3.45 (0.66)	3.50 (0.17)	97.0	0.037	0.617
Privacy and security	15 (100)	3.80 (0.82)	4.00 (0.22)	61.5	0.009	0.864
Response time	15 (100)	4.33 (0.62)	4.00 (0.16)	105.0	<0.001	1.000

Note. H_a_ μ ≠ 3.

**Table 4:** Results from the uMARS (N = 15).

	Participants, *n* (%)	Values, Mean (SD)	Values, Median (Range)	*W*	*p* Value	r
Engagement	15 (100)	3.35 (0.80)	3.60 (0.21)	66.0	0.160	0.451
Information	13 (86.67)	4.23 (0.48)	4.25 (0.13)	91.0	0.002	1.000
Subjective items	15 (100)	3.30 (0.61)	3.50 (0.16)	52.5	0.089	0.591
Perceived impact	15 (100)	3.47 (0.81)	3.67 (0.21)	90.0	0.093	0.500

Note. H_a_ μ ≠ 3.

**Table 5:** Main positive and negative aspects emerged from user interviews.

	Communication Style and Tone	Module Structure	Personalization	Perceived Usefulness/Effectiveness
Positive	“It gave you time. In several moments it explicitly said things like ‘take all the time you need’ or ‘ I know this takes time’… I really appreciated this, because it didn’t pressure you to respond or rush through the session.” —midwife	“I appreciated when the chatbot included exercises within the session, rather than leaving them to be done independently, because it made it easier to stay engaged throughout the interaction.” —usability expert	“I found it useful to be able to choose whether to repeat or skip certain parts, such as the myths. Having the option to decide based on my needs made the interaction feel more adaptive.”—psychologist	“I really appreciated the aim and overall meaning of the intervention. I think this kind of support can be very useful, especially in helping women see things from a different perspective and find more strength in facing this period.”—expecting mother
Negative	“I believe the ‘warmth’ of the intervention could be ameliorated—it really felt like you were talking with a chatbot sometimes… I noticed it repeated some answers—things like ‘Sad moments happen to everyone”—which were fine, even correct you might say, but after reading it once again it felt empty.” —psychologist	“Some sessions felt too long, and at times I interrupted them and continued later, or even forgot to resume them completely.” —usability expert	“Even if you could give different answers, the interaction always brought you back to the same path, so it didn’t really feel personalized.”—expecting mother	“Some of the messages felt quite theoretical and didn’t really push me to put things into practice.”—expecting mother

## Data Availability

The datasets used and/or analysed during the current study are available from the corresponding author on reasonable request.

## Supplementary Materials

The following supporting information can be downloaded at: https://www.mdpi.com/article/10.3390/bs16040585/s1, Table S1: TIDieR checklist (includes testing procedure); Table S2: Post hoc mapping of study indicators to TFA and RE-AIM domains.

## Abbreviations

The following abbreviations are used in this manuscript:

ACT	Acceptance and Commitment Therapy
NICE	National Institute for Health and Care Excellence
ORBIT	Obesity-Related Behavioral Intervention Trials
uMARS	User Version of the Mobile Application Rating Scale
BUS-11	Chatbot Usability Scale
SD	Standard Deviation

## Appendix A

**Table A1:** List of variables and subvariables investigated and related items used.

Variable	Subvariables	Items
Communication	empathy and listening skills	judgmental—welcoming, passive listening—active listening, alarming—reassuring, indifferent—sensitive, cold—warm
	fluency and fluidity	non-sliding—sliding
	chatbot interaction	boring—engaging, inefficient—efficient, slow—fast, heavy—adequate
	vocabulary	abstruse—understandable, technical—common
Module structure	interaction duration	long—short, challenging—easy
Material	videos	unpleasant—pleasant, stressful—relaxing, hindering—helpful, useless—functional
	infographics	unimaginative—creative, hindering—supportive, unpleasant—pleasant
Content	content evaluation	boring—interesting, soporific—stimulating, of little value—of great value
	clarity of content	confused—clear, abstruse—understandable

## Appendix B

**Table A2:** List of topics and related questions used during the semi-structured interview.

Topic Investigated	Questions
Interaction	1.1 How did you feel about the interaction with DEMETRA? Were there enough alternatives among the replies to DEMETRA? 1.2 What was the feature of the interaction that you liked the most? And the one you liked the least? 1.3 Have you ever made the mistake of clicking the answer button on a question? Have you wished you could have gone back?
Contents	2.1 If something was not clear to you at first, do you find there is then a way to investigate the topic further? 2.2 Is the mode of communication (length of sentences, terms used, etcetera) appropriate for the content?
Engagement	3.1 Was the communication with the chatbot engaging? Did it entice you to get involved and be consistent in the activities it recommended?
General	4.1 Do you have any concerns? Do you have any criticism? 4.2 When would be the ideal time to take this intervention for a pregnant woman? 4.3 Overall, was the intervention personalized for you? Give a level of personalization from 0 to 10.
