# Supplementary material for: Digital Acceptance and Commitment Therapy for Lifestyle Change in Overweight Pregnant Women: A Feasibility Pilot Study
Source: Behav Sci (Basel). 2026 Apr 14;16(4):585. doi: 10.3390/bs16040585 (PMC13113308; doi:10.3390/bs16040585)
Supplement: Supplementary file 1 [file behavsci-16-00585-s001.zip › behavsci-4189112-Supplementary.pdf]

Supplementary Materials

Table S1. TIDieR checklist (includes testing procedure).

N	Item	DEMETRA (intended intervention)	Prototype testing procedure (as implemented in this study)
1	Brief name	DEMETRA (rule-based virtual assistant/chatbot)	DEMETRA prototype tested with stakeholders
2	Why (rationale/ theory/goal)	ACT-informed psychoeducational and motivational intervention aiming to enhance psychological flexibility to support healthier lifestyles (nutrition and physical activity) during pregnancy; not focused on intentional weight loss during pregnancy. Content architecture informed by The Diet Trap (Lillis et al., 2014), Healthy Habits Suck (Lee-Baggley, 2019), and ACT Matrix resources (Polk & Prevedini, 2017; Polk et al.).	Same rationale; evaluation purpose framed as formative mixed-methods assessment of acceptability, usability, and perceived relevance prior to efficacy testing (ORBIT Phase 1 – Design).
3	What (materials)	Dialogue scripts adapted into concise modules; multimedia resources (videos, audio, images) created to convey key information accessibly.	Sessions delivered via WhatsApp providing readable and watchable content, the same as those involved in the intended intervention.
4	What (procedures)	Six-session program, once a week; DEMETRA initiates interaction; provides psychoeducational content and prompts self-reflection through targeted questions; end-of-session exercise instructions for the subsequent week. Users cannot submit free queries; interaction proceeds through predefined options/branches.	Six-session program. Participants received opening session and instructions by email; then received subsequent sessions according to accelerated schedule. Participants interacted via close- and open-ended responses to proceed; participant answers were neither collected nor analysed in this prototyping phase. After testing, participants completed questionnaires (email) and semi-structured interview (audio-recorded).
5	Who provided	Delivered by rule-based chatbot. Intervention materials developed by multidisciplinary team led by AEN (psychologist/researcher) with psychologists, ACT experts, nutritionists, communication and IT experts; biweekly meetings; ACT expert coordinated group discussions to ensure shared understanding.	Same; iterative tests included professionals and end users. Interviews conducted by a psychologist.
6	How (mode of delivery)	Planned digital delivery by a mobile application guided by DEMETRA (stated as future availability once perfected).	Delivered via WhatsApp during prototype evaluation. Message pacing not controllable on Landbot (no/low-code chatbot building platform).
7	Where (setting)	Intended for remote use during pregnancy (digital tool). Recruitment based on inclusion/exclusion criteria; all materials and interviews in Italian; data pseudonymized.	Remote, on participants' own devices; recruitment via convenience sampling; all materials and interviews in Italian; data pseudonymized.

8	When and how much (dosage/ intensity)	Scheduled to span six weeks. Each session lasts ~15 minutes. On designated day, DEMETRA delivers material; exercise to be completed in subsequent week.	Scheduled to span two weeks. Participants received the intervention every 48 hours. Sessions lasted an average of 15 minutes.
9	Tailoring	Initial session: users choose preferred day of the week and time slot (morning/afternoon/evening). Branching during the intervention: review previous session; complete exercise immediately or later; other branches "and so on".	Same tailoring features available during testing.
10	Modifications	Developed iteratively; content drafted and refined through iterative cycles into six-session program; multimedia added.	Same iterative evaluation stages.
11	How well (planned fidelity)	Rule-based scripted structure supports standardised delivery; six-session thematic structure defined (Table 1).	Same. No additional fidelity monitoring procedures.
12	How well (actual fidelity/adherence)	Not assessed as real-world adherence over 6 weeks in this formative phase.	Completion: 16 participated; 15 completed questionnaires; 16 interviews completed. No session-by-session completion metrics.

Table S2. Post hoc mapping of study indicators to TFA and RE-AIM domains.

Framework	Domain/construct	Indicators collected in this study (instrument / source)	Notes / gaps (planned in future work)
**TFA**	Affective attitude (liking)	Qualitative interviews; uMARS (subjective quality); BUS-11 (overall appraisal/usability-related satisfaction)	Not assessed longitudinally
**TFA**	Burden (perceived effort)	Semantic Differential (perceived duration/effort-related items); qualitative interviews (pacing/length)	No long-term burden with real-world weekly schedule
**TFA**	Intervention coherence (understanding)	uMARS (information quality); qualitative interviews (clarity/comprehension)	No dedicated TFA coherence scale
**TFA**	Perceived effectiveness	uMARS (perceived impact); qualitative interviews (usefulness/expected benefit)	No behavioural/clinical outcomes in this phase
**TFA**	Self-efficacy	Qualitative interviews (perceived ability to perform exercises)	No quantitative self-efficacy measure

**TFA**	Ethicality (fit with values/appropriateness)	Qualitative interviews (tone/appropriateness in pregnancy; non–weight-loss emphasis)	Not systematically quantified
**TFA**	Opportunity costs	Qualitative interviews (time/attention trade-offs)	Not systematically quantified
**RE-AIM**	Reach	Recruitment description and sample characteristics	Not estimated in target population
**RE-AIM**	Adoption	Inclusion of clinicians/professionals as stakeholders (qualitative + quantitative feedback)	No organisational adoption testing
**RE-AIM**	Implementation	Dose described (intended 6-week vs accelerated 2-week testing); qualitative feedback on delivery constraints	No fidelity monitoring, cost evaluation, or workflow assessment
**RE-AIM**	Maintenance	—	Not assessed (out of scope for ORBIT Phase 1); planned in later phases

Note. This mapping is provided to enhance transparency; the study was not prospectively designed to operationalise all TFA/RE-AIM constructs.
